# The ethical imperative to honor autistic clients’ autonomy in mental health treatment

**DOI:** 10.3389/fpsyt.2023.1259025

**Published:** 2023-09-25

**Authors:** Alana J. McVey, Katherine Jo Glaves, Samantha Seaver, Karís A. Casagrande

**Affiliations:** ^1^Department of Psychiatry and Behavioral Sciences, University of Washington, Seattle, WA, United States; ^2^Autism Center, Seattle Children’s Hospital, Seattle, WA, United States; ^3^Antioch University Seattle, Seattle, WA, United States

**Keywords:** autism, stigma, mental health, treatment, client autonomy

## Abstract

Autistic adolescents and adults commonly experience mental health concerns; however, mental health clinicians may hold implicit stigmatizing views of autism that contribute to case conceptualization and treatment goal setting that align more with caregivers’ than clients’ goals. This impingement on client autonomy is concerning, problematic, and potentially harmful for autistic clients who are of an age to set their own treatment agenda regardless of co-occurring intellectual disability and/or language delays. An application of the shared decision-making framework, an evidence-based tool for promoting client autonomy, can help to avoid these challenges in treatment. In this perspective, we use a case vignette as an anchor for discussing the imperative of honoring autistic clients’ autonomy in mental health treatment and guiding shared decision-making to reduce stigma, promote autonomy, and increase collaborative care for autistic clients in mental health treatment.

## 1. Introduction

Autonomy is a key principle in mental health practitioners’ ethical codes (1–4). Yet, clinicians may hold implicit stigmatizing views of autistic adolescent and adult clients, especially those with co-occurring intellectual disability and/or language impairment. This harmful perspective may contribute to the assumption that these clients are unable to direct their own treatment and result in deriving treatment goals from caregivers’, rather than clients’, presenting concerns. This can be problematic, as autistic youth and their caregivers often have different goals for their future (5). This bias results in a disregard of the autonomy of these clients and can cause harm when case conceptualization, treatment goals and planning, and components of treatment are not well-matched to a client’s wants, needs, or preferences and desires for their future. Additionally, thwarting an autistic client’s autonomy may contribute to ongoing mental health challenges by contributing to a client’s lack of agency and increasing their internalized ableism, infantilization, and learned helplessness. While working collaboratively with an autistic client in goal setting and treatment planning may require additional time and creativity, not doing so poses a significant ethical concern and can impair a client’s ability to make progress, as they will likely be less willing to work towards goals they are not invested in meeting. Conversely, when clinicians work collaboratively with clients, clients are more likely to experience a sense of allyship with clinicians, an increased internal locus of control, and greater intrinsic motivation for skill building, leading to improved clinical outcomes.

In this perspective, we bring together our expertise as mental health providers and clinical researchers, as well as our lived perspectives as non-autistic neurodivergent and neurotypical allies of autistic people to discuss the imperative of honoring autistic clients’ autonomy in mental health treatment. We acknowledge that mental health clinicians may themselves be autistic or otherwise neurodivergent, and while our guidance may be helpful to all, it is most intended for neurotypical clinicians. We use a case vignette as an anchor for discussing the imperative of honoring autistic clients’ autonomy in mental health treatment and guiding decision-making. This vignette has been constructed by combining the experiences of numerous clients into one hypothetical case.

*Jacob, a 16 years-old Black cisgender, heterosexual young man, is autistic, has a mild intellectual disability, and is clear about what he wants from therapy*. “*I want to have a girlfriend*,” *he says. His parents tell you that that is not their goal: they want Jacob to be* “*less disruptive*.” *Jacob’s parents state that Jacob will* “*scream and shout*” *when he is upset or denied something he wants. These behaviors occur more at home than in other settings. When you try to talk to Jacob about his desire to date, his parents interject to tell you that Jacob does not need a girlfriend since*, “*it wouldn’t be appropriate for him to date*.” *In a one-on-one session, Jacob tells you that he is* “*really excited*” *to have sex, but when he got condoms from his doctor (following a conversation that Jacob initiated about safe sex), his parents took them away, saying pre-marital sex is a sin in their Christian faith*.

This scenario forces us to confront the complex issue of client autonomy when caregiver and client goals conflict. Jacob’s parents are focused on his “disruptive” behavior. To Jacob, having a girlfriend is a meaningful goal. Many questions come to mind when considering how to address this conflict. How do we conceptualize the presenting concerns? With whose goals do we align? Who decides what therapy goals are appropriate and legitimate? What happens when an adolescent client and their caregivers disagree on goals for therapy?

We propose that supporting autonomy through the evidence-based framework of shared decision-making (6–8) can guide clinicians in working effectively with autistic adolescents and adults presenting for mental health treatment. Following Simon et al. (7) steps, this involves: (1) recognizing that a decision needs to be made, (2) identifying partners in the process as equals; (3) stating the options as equal; (4) exploring each person’s understanding and their expectations; (5) identifying preferences; (6) negotiating options/concordance; (7) sharing the decision; and (8) evaluating decision-making outcomes. Shared decision-making relies on collaboration between clinicians, clients, and family members. Importantly, it centers the client’s goals, preferences, and identities.

## 2. Centering autonomy

Greater levels of client autonomy are related to improved clinician-client communication quality (9, 10) and increased motivation, treatment participation and satisfaction, and quality of life (9–12). Decreased client autonomy, conversely, has been associated with higher levels of depression and anxiety symptoms (13). Pelletier et al. (12) identify that supporting a client’s autonomy allows them to experience their behavior as caused by their own motives and goals (internal locus of causality). When clients perceive clinicians as more controlling and less supportive of their autonomy, clients report less motivation to “buy in” to treatment (12). When it comes to autistic clients, some researchers have spoken *against* promoting autonomy in therapeutic relationships (14, 15), despite literature that demonstrates substantial overlap between the needs of autistic and allistic clients in therapeutic relationship building (16). Outdated viewpoints such as this perpetuate the myth that autistic clients do not deserve autonomy in treatment and demonstrate the paternalistic attitudes that clinicians have historically taken toward autistic people, setting the stage for further exclusion of neurodivergent clients from models of therapeutic alliance. We propose that the application of shared decision-making, integrated with more progressive recent therapeutic considerations, supports a clinician in setting aside their own biases to meet their client as an equal in the treatment process.

Kinsella (17) provides a humanistic perspective and argues that it is a clinician’s ethical duty to foster clients’ autonomy. He states that an ethically-grounded practice requires believing that clinicians can nurture clients’ autonomy by being adaptive and supportive of each client’s strengths and needs. This can be done, not only by fostering autonomy where it exists, but also by promoting it where it is lacking. Additionally, Chapman and Botha (18) propose a neurodivergence-informed psychotherapeutic framework, arguing against default normalization and pathologization and for neurodivergent prosperity. One of the three themes they propose is for neurotypical clinicians to cultivate “epistemic humility”—the ability to change one’s assumptions and biases through critical reflection—in order to foster a collaborative approach within the client-clinician dyad and respect the client’s lived experience.

Bearing this in mind, when faced with Jacob’s parents’ requests to reduce his “disruptive behavior,” we might ask ourselves, “What is happening, internally, for him when he behaves this way?” Jacob is likely distressed when he is “disruptive;” his behaviors can be seen as communicating that distress to his parents. To ignore Jacob’s internal experiences is to overlook his valid frustrations, which disregards his personhood and autonomy.

As the name suggests, shared-decision-making centers around engaging the client in decision-making about their own treatment—a direct application of supporting client autonomy. The clinician empowers the client to make decisions by providing them with options, establishing and validating their expertise, and actively working to address misunderstandings when they arise. In Jacob’s case, we would suspend the assumption about his inability to direct his own care due to his diagnoses of autism and intellectual disability. Rather than approaching Jacob’s case from his parents’ perspective of disruptive behaviors, a shared decision-making approach would support Jacob in communicating his perspective. By addressing the differing conceptualization of the presenting concern, we create the opportunity to discuss family dynamics and provide psychoeducation on appropriate teen autonomy and safe sex practices.

### 2.1. Supporting relatedness

While we may think of autonomy as pertaining to an individual, Kinsella (17) rethinks it as a reciprocal and relational process. He emphasizes the importance of a clinician replacing paternalism with a more egalitarian “relatedness.” Chapman and Botha (18) emphasize that neurodivergence-informed therapy relies upon a relational model of mental health. From this view, the challenges an autistic person experiences are due to dysfunctions in the relationship or differences in communication, rather than dysfunction that is intrinsic to the autistic client. In Jacob’s case, we can reflect on our own biases, neurotype, and communication styles to understand our role in relational dysfunctions that may occur in therapy, and we can view the concerns he and his parents raise as occurring within the context of their family system; that is, between Jacob and his environment, rather than as a flaw within Jacob himself.

Reconceptualizing the conflict between Jacob and his caregivers around the issue of premarital sex as relational can be beneficial in understanding how to engage in shared decision-making. For example, it is important to assess Jacob’s feelings about premarital sex in terms of *his* faith—we should not assume Jacob shares his family’s faith. In addition, if Jacob shares his family’s faith, in which premarital sex is immoral, and Jacob’s parents believe he cannot date or get married, Jacob may be in a double-bind: wanting sex, not considered competent to be married, but in a faith system that holds marriage is the only way one can have sex. This double-bind, a term from strategic family therapy (19), can cause a person to feel anger, rage and resentment as their autonomy is being denied. These may be some of Jacob’s internal experiences when he exhibits “behavior problems.”

Jacob’s parents seem to be vocalizing distress by the idea of him dating; they find it “inappropriate.” Almost all 16 years-old, including autistic and developmentally delayed 16 years-old, have sexual feelings [e.g., (20)]. If Jacob’s parents deny him appropriate teen autonomy, Jacob may push back by acting out, which may cause Jacob’s parents to see him as younger than his age. This circle of events occurs frequently in families with autistic teens and young adults, as parents fail to see their adolescent or young adult’s behavior as age-appropriate bids for autonomy, instead viewing them as “childish” outbursts. Jacob’s parents may be trying to protect Jacob from the risks of dating, but there is a dignity to risk taking—one that people with intellectual disabilities are often denied. “Perske (21) wrote, ‘We have yet to completely evaluate what we do to the human dignity of (people with intellectual disabilities) when such relationships are denied.’ To be a person is to strive and, at times, to fail. We deny personhood to those who we do not allow to fail” (22), p. 311.

Applying a model of shared decision-making inherently supports a relational and collaborative approach to care. Simon et al. (7) specifically propose steps to shared decision-making that address the concerns about relatedness discussed above, including developing a greater understanding of expectations and exploring the client’s preferences, concerns, and goals. Shared decision-making invites ongoing communication from all parties to explore miscommunications or conflicting perspectives throughout treatment. It also creates a space to discuss the roles and responsibilities of all parties involved in treatment. Jacob’s desire to date is normative and healthy, not pathological. As his clinician, we might offer psychoeducation on healthy relationships, how to set and hold to boundaries, or other information to support a client dating in healthy ways, but we would not take steps to deny Jacob’s autonomy to date.

### 2.2. Validating identity

Race and ethnicity play an important role in the quality of the client-clinician relationship and treatment outcomes. Barzargan and colleagues (23) found that non-Hispanic Black and Hispanic clients had higher medical mistrust than non-Hispanic white clients of racial and ethnic minority backgrounds who report less respect and dignity in their treatment are less likely to follow medical recommendations (24). Trust, respect, dignity, and client-centeredness—treatment factors associated with racial and ethnic differences in the client-clinician dyad—are imperative to bolstering client autonomy.

Jacob’s intersecting identities as a Black autistic young man are crucial for us to consider, especially when we do not share his identities or lived experiences. Given racism and racial stereotypes, Jacob’s parents may have legitimate fears around him being perceived as the aggressor in a sexual relationship; to ignore this possibility is to place Jacob in danger. We could address this concern with Jacob by teaching him about consent, gaining clarity, and not making assumptions with romantic partners. Jacob has demonstrated responsible behavior about sex by seeking condoms and information about safe sex; highlighting this to his parents as a strength may help to alleviate some of their concerns.

Regarding his neurodivergent identity, Jacob and his parents may or may not see autism as something to celebrate. The common discourse about autism from a medicalized view may contribute to Jacob’s parents’ stigma and Jacob’s own internalized stigma. His parents may wish to impose their treatment goals on Jacob out of fear that he cannot make appropriate decisions. Autism can be conceptualized as a “neurominority” (18)—an identity that is a source of pride, belonging, and competence. Connecting Jacob with resources and role models to support autistic joy and pride (18) may support his autonomy development, mental health, and wellbeing (25, 26). Additionally, educating Jacob’s parents about autistic identity from a neurodiversity perspective may help to reduce stigma and set the stage for them to support Jacob in becoming an autonomous adult.

Without this neurodivergence-affirming lens, clinicians would be unable to engage in shared decision-making as they need to perceive their clients as equal partners in the process. Jacob showed mature judgment in seeking contraception from his doctor; noticing and praising that choice will foster his autonomy and help Jacob see himself as capable of making decisions that support his wellbeing. Jacob’s parents might likewise be able to see how Jacob was being responsible by asking for contraceptives, even if they do not agree with Jacob having sex.

## 3. Conclusion

Promoting client autonomy is a key principle of care across ethics codes for various mental health practitioners (1–4). Most states allow minors to consent to their own outpatient mental health treatment and many have additional provisions of confidentiality and limited disclosures to legal guardians (27). While this level of autonomy and control over treatment is often the default for neurotypical minors entering therapy, autistic adolescents and adults, especially those with co-occurring intellectual disability and/or language impairments, are often not given the same opportunities. To promote the best possible outcomes in line with our ethical duties as mental health professionals, it is critical that we support the autonomy and dignity of risk of autistic clients.

For Jacob, we can promote autonomy, address the bias toward paternalization, and increase the quality of collaboration by following a model of shared decision-making [e.g., (6–8)]. By engaging clients directly in making choices about their treatment, respecting their experiences and intersecting identities, and working collaboratively across clients, clinician, and caregivers, outcomes for clients like Jacob can change dramatically.

*Jacob’s clinician laid out the conflict between Jacob and his parents and the fact that treatment goals were needed; Jacob and his parents agreed this was true and that they all wanted something to change*.

*The clinician then explored with Jacob and his parents the meaning of their goals; Jacob’s parents were able to discuss their worries for Jacob’s future and how his outbursts lead them to fear for his safety, particularly as Jacob is a Black male. Jacob needed the context of this worry explained, but when it was, he began to understand his parents’ fears*.

*Jacob was likewise able to tell his parents he had always dreamed of being* “*married like you*” *which touched Jacob’s parents deeply. Jacob likewise explained that he knew, from school, that sex was important to do* “*right*,” *and that safe sex was* “*right sex*.” *Jacob’s parents were able to see the importance of dating to Jacob. They expressed the preference that Jacob wait to have sex until marriage, but also stated they were glad he was* “*thinking about safety*.” *Jacob, for his part, was able to agree that less conflict at home would be good*.

*Jacob and his parents negotiated an agreement: Jacob and his parents would work with the clinician to reduce conflict at home. Jacob’s parents agreed that Jacob could date if he found a girlfriend*.

*The end treatment goal prioritized reducing conflict. Jacob, understanding his parents’ fears for his life, was more willing to work on reducing outbursts by using his coping skills, saying* “*I don’t want to get shot*.” *The treatment goals were reviewed after six months; at that time, Jacob’s parents reported* “*less than one outburst a week*” *and Jacob was planning to ask a girl from his class to a church picnic*.

Furthermore, it is important to understand the role of identity in challenges to client autonomy. For Jacob, his various intersecting identities as a Black, cisgender, heterosexual autistic teen with an intellectual disability from a family with a religious background affect the way in which he is perceived and how his family or clinicians choose to interpret his behaviors. Historically, being autistic has been cited as a reason for excluding clients from shared decision-making regarding their care [e.g., (14, 15)]. Instead, we urge clinicians to engage in collaborative care that honors the autonomy and dignity of autistic clients. The shared decision-making model reminds us to respect the expertise and lived experience of our clients as equals in therapy, creating multiple points at which to engage in conversation about goals and motivations for treatment. This model helps ensure we are centering our clients’ autonomy in a way that is relational and identity affirming.

## Data availability statement

The original contributions presented in the study are included in the article/supplementary material, further inquiries can be directed to the corresponding author.

## Author contributions

AM: Conceptualization, Writing – original draft, Writing – review & editing. KG: Conceptualization, Writing – original draft, Writing – review & editing. SS: Writing – original draft, Writing – review & editing. KC: Conceptualization, Writing – original draft, Writing – review & editing.
